# Perception of farmers about endometritis prevention and control measures for zero-grazed dairy cows on smallholder farms in Rwanda

**DOI:** 10.1186/s12917-020-02368-6

**Published:** 2020-06-05

**Authors:** Pascal Nyabinwa, Olivier Basole Kashongwe, Claire d’Andre Hirwa, Bockline Omedo Bebe

**Affiliations:** 1 Rwanda Agriculture and Animal Resources Development Board, P.O; Box 5016, Kigali, Rwanda; 2 Department of Animal Sciences, Faculty of Agriculture, Egerton University, P.O; Box 536, Egerton, Kenya

**Keywords:** Herd health, Best-worst scaling choice, Extension messages, Management interventions, smallholder farmers

## Abstract

**Background:**

Endometritis is a prevalent uterine disease in postpartum cows. The disease reduces fertility performance and milk yield, and subsequently, productivity and profitability of dairy farms. The reduction in performance is associated with considerable economic losses on dairy farms. Smallholder farmers are likely to incur considerable economic losses from the disease where they lack knowledge of effective prevention and control measures for the disease. This study used farmer’s perspectives to determine the effectiveness of different management interventions (MIs) for endometritis prevention and control on smallholder farms in Rwanda practicing dairy zero-grazing. The best-worst scaling (BWS) choice method was applied that relied on past 1 year recall data obtained from 154 farmers. These farmers were identified through snowball sampling in a cross-sectional study.

**Results:**

Of the 20 MIs evaluated, 12 scored highly for effectiveness. The top four most effective are: avoiding sharing equipment with neighbouring farms (45.5%), consulting animal health service provider about disease treatment (31.8%), keeping cows in a clean and dry shed (26.7%), and selecting sires based on calving ease (26.6%). The MIs considered least effective were: maintaining clean transition cow housing (35.1%), removal of fetal membrane immediately after passing (33.1%), disinfecting the equipment used in calving assistance before and after use (32.5%), and selecting sires with low percent stillbirths (29.2%).

**Conclusion:**

This study has demonstrated the application of BWS object case method in understanding the MIs that farmers consider are most effective in the prevention and control of endometritis disease in the dairy herds. The MIs are on-farm biosecurity and hygiene, seeking veterinary services for disease treatment and selecting sires for ease of calving. These MIs should be considered for prioritization in extension services and research to continuously improve and enhance their practical application on smallholder dairy farms.

## Background

Dairy production is a major component in the livestock sector in Rwanda. The dairy subsector is an essential source of livelihood to over 80.0% of households involved directly or indirectly throughout the agricultural value chain [1]. The dairy subsector contributes 28.0% to the agricultural Gross Domestic Product (GDP) and 4.0% to the national GDP [2]. Rwanda has an estimated cattle population of 1,340,792, of which 45.0% are indigenous cattle, 33.0% are dairy crossbreds, and 22.0% are pure dairy breeds [3]. The dairy crossbreds and pure dairy breeds are of the Friesians, Jersey, and Fleckvieh breeds. Among the smallholder dairy farms, those practicing zero-grazing hold the majority (92.0%) of the cattle population and supply the bulk of the domestic milk market demand. However, the supply has not satisfied the local demand. The per capita milk consumption estimates by the Rwanda Livestock Master Plan [1] is 63.0 l per person per annum. An increase of 3.5 fold would be necessary so as to achieve per capita consumption threshold of 220 l recommended by the Food and Agriculture Organization of the United Nations [4]. The low per capita milk consumption is to a large extent due to low productivity of the national herd, and this is attributable to suboptimal fertility performance of zero-grazed cows in smallholder dairy farms [5–7].

One disease associated with suboptimal fertility, though often unnoticed, is endometritis disease [8, 9]. Endometritis is a disease of dairy cows occurring between the 21st and 90th days postpartum. The contamination of uterus by endometrial microbiota occurs at all stages of the reproduction cycle [10], but the majority of cases are found mostly during the first 2 weeks of postpartum [11]. This contamination is attributed to the fluctuation and expansion of the microbial community diversity after calving. The reason for this is because of the dilation of physical barriers such as vulvar sealing, vestibule-vaginal constriction, the cervix, cervicovaginal mucus secretion, and the epithelial barrier. These allow contamination and colonization of the genital tract with pathogenic bacteria from the environment, skin, faeces, and vagina [12]. These pathogenic bacteria such as *Escherichia coli*, *Fusobacterium necrophorum*and *Staphylococcus aureus* are the common causes of endometritis in dairy cattle [13]. The di, sease results in considerable economic losses through the reduction in production and fertility performance, culling of cows, and veterinary costs [8, 9].

Good management practices in the pre-and postpartum period can minimize or even avoid cow uterine infections and prevent the prevalence of endometritis disease [14]. In contrast, suboptimal management of transition cows exposes susceptible cows to postpartum uterine diseases in which endometritis is of importance [10, 12]. Sadly, effective treatment options for endometritis remain limited, yet the disease can persist even after treatment and recovery [15, 16]. This means that treating the condition is not a solution; it is necessary to implement effective prevention and control measures [12, 14]. Management interventions (MIs) that prevent the introduction and reduce the spread of disease-causing agents into and off the herd are critical components of the herd health program [17–19].

The MIs could significantly minimize endometritis incidences and consequently improve animal welfare and increase productivity and profitability of dairy herds [18]. This is supported by observations that improved extension service and advisory support in the pre- and postpartum periods improve the prevention and control of endometritis in the dairy herds [20]. In extension service delivery, farmers are essential in implementing MIs and evaluating the effectiveness of the different MIs for disease prevention and control [18, 21].

The best-worst scaling (BWS) choice is a preferred technique to gather opinions from different experts on the effectiveness of varying biosecurity measures on dairy farms [21, 22]. The BWS has been used in market research [23]; human health [24]; agriculture [25], and livestock management science [26, 27].

The literature search revealed no application of BWS choice to endometritis management studies. Empirical evidence on how dairy farmers perceive the effectiveness of MIs for endometritis prevention and control is yet to be documented. However, BWS holds great potential in determining effective MIs from farmers’ perspectives; they are the implementers. Advances in this knowledge gap would be informative to actors in the dairy sector towards reducing the prevalence rate of endometritis disease in the dairy herds. In particular, extension service and farmers stand to benefit from the immediate application of effective MIs.

In Rwanda, smallholder dairy zero-grazing is a priority development intervention towards hunger eradication and attaining food and nutrition security [1]. High prevalence of endometritis in the herd could, however, impede the achievement of these development goals due to the economic loss associated with the disease. For this reason, this study evaluated the opinion of farmers about the effectiveness of different MIs for endometritis prevention and control under field conditions in Rwanda. The research will inform prioritization of MIs in extension service and for on-farm implementation.

## Results

### Socio-economic characteristics of the dairy farmers

The socio-economic characteristics of the sample farmers in the study area are presented in Table 1. The sampled farmers were between 25 and 85 years old and on average, were of middle-aged (41.5 ± 1.1 years) with an average of 9.6 ± 0.5 years of dairy farming experience. The majority were males (71.4%). About half had attained primary level education (48.7%) while a few had attained secondary (14.9%) or university (2.6%) level education. However, over a third had not acquired any formal education (33.8%). The family size was, on average, 5.0 ± 0.1 members in a household. By poverty classification of the Rwanda government, farmers in the category of poor (63.0%) dominated over those in the category of wealthy farmers (28.6%). On average, a household kept a herd of less than 3 cattle on a farm of less than 4 acres (Table 1).

**Table 1:** Socio-economic characteristic of the sample smallholder dairy farmers (*n* = 154)

Variables	Frequency (%)	Mean ± S.E.	Min – Max
Gender			
Male (%)	71.4		
Female (%)	28.6		
Educational level			
No schooling (%)	33.8		
Primary (%)	48.7		
Secondary (%)	14.9		
University (%)	2.6		
Poverty level			
Very poor (%)	8.4		
Poor (%)	63.0		
Rich (%)	28.6		
Age (years),		41.5 ± 1.1	25–85
Dairying experience (years)		9.6 ± 0.5	1–25
Household size (number)		5.0 ± 0.1	1–9
Herd size (number)		2.9 ± 0.8	1–4
Farm size (acres),		3.8 ± 0.1	0.6–7.4

The herds were predominantly crossbreeds (63.6%) with some pure breeds (29.0%), and indigenous cattle breeds (7.4%) kept to provide milk for domestic consumption, sale, and to provide manure for fertilizing the farms. The cows were kept in zero-grazing housing units, on the cut-and-carry feeding system, with over half servicing with bulls (53.9%). At the time of this study, the breeding services were at a cost: $5.6 per service when using artificial insemination service and $3.3 per service when using bull service. Dairy farmers sourced animal health services (ANHS) from veterinarians (VETs) (39.6%), community-based animal health workers (CAHWs) (37.7%), and local traditional herbalists (LTHs) (22.7%). The animal recording was not practiced (78.6%) or incomplete (21.4%).

The flooring of cattle shed was typically earthen (90.3%), and only a few had concrete (9.7%). Bedding materials were natural green grasses (85.5%), and the leftovers or waste feeds from feeding troughs (14.5%) (Table 2). The frequency of removal of leftovers or waste feeds was, on average, twice a week.

**Table 2:** Herd characteristics in the study area (*n* = 154)

Characteristics	Level	Frequency (%)	Chi-square test Sign
Cattle shed flooring	Earthen	90.3	***
	Concrete	9.7	
Types of cattle shed	With a roof	65.6	***
	Without a roof	34.4	
Breeding services	Artificial insemination service	46.1	NS
	Bull service	53.9	
Herd record keeping	Incomplete	21.4	***
	Not practiced	78.6	

*NS* Not significant (*p* > 0.05), ***p < 0.001

### Effectiveness of the management interventions

**Fig. 1:**
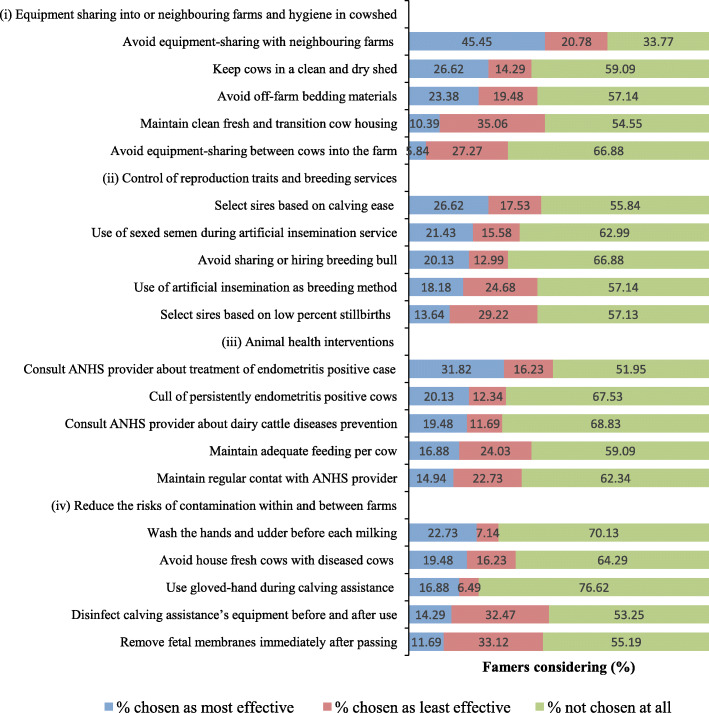
Best-worst percentages of farmer’s opinion on the effectiveness of 20 management interventions towards endometritis on dairy farms (*n* = 154). *ANHS = animal health service

Figure 1 shows the percentage of MIs that farmers considered the most effective, least effective, and not considered the most or least effective. In decreasing order of probability of being considered most effective, the top four MIs were: avoiding equipment-sharing with neighbouring farms (45.5%), consulting ANHS provider about the treatment of endometritis positive case (31.8%), keeping cows in a clean and dry shed (26.7%), and selecting sires based on calving ease (26.6%). The MIs considered least effective were: maintaining clean transition cow housing (35.1%), removal of fetal membrane immediately after passing (33.1%), disinfecting calving assistance’s equipment before and after use (32.5%), and selecting sires with a lower percentage of stillbirths (29.2%). The MIs not considered to be effective were: use of gloved-hands during calving assistance (76.6%), washing the hands and udder before each milking (70.1%), consulting ANHS providers about dairy cattle diseases prevention (68.8%), and cull of persistently endometritis positive cows (67.5%). The study revealed that neither dairying experience, poverty level, education level, nor the source of ANHS providers influenced (*p* > 0.05) the considerations by farmers about the MIs.

**Fig. 2:**
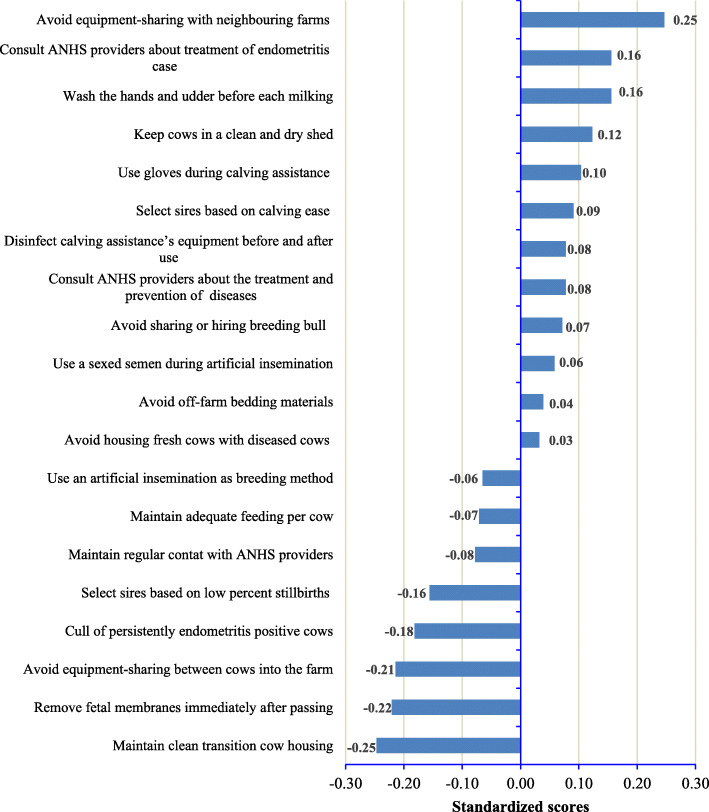
Effectiveness scores for the 20 management interventions (MIs). *ANHS = animal health service

The standardized scores illustrated in Fig. 2 represent the computed effectiveness scores assigned to each MI. The y-axis represents the effectiveness scores of all 20 MIs that were examined. The right of the x-axis shows the MIs that were scored highly for effectiveness whereas the left of the x-axis represents the MIs that were scored low for effectiveness.

Of the 20 MIs, 60.0% (*n* = 12) were scored highly for effectiveness, and these are located in the upper right-hand quadrant. These MIs belong to equipment-sharing between cows within farms and/or with neighbouring farms and hygiene in a cowshed (group 1 of MIs: 02, 04, and 10), control of breeding services (group 2 of MIs: 05, 14 and 19), animal health interventions (group 3 of MIs: 06 and 07), and reduce the risks of contamination within and between farms (group 4 of MIs: 03, 09, 17 and 20). Based on standardized scores, 20, 15, 15, and 10% of MIs were considered as the most effective MIs in group 4, 1, 2, and 3, respectively. Avoiding equipment sharing with neighbouring farms (MI 02), consulting ANHS provider about the treatment of positive endometritis case (MI 07), and washing the hands and udder before each milking (MI 20) were the perceived most effective MIs. However, there were no significant differences (Chi-square = 1.583, *p* = 0.663) found among the four groups of MIs.

The MIs scored low for effectiveness were: maintaining clean transition cow housing (MI 12), removing fetal membranes immediately after passing (MI 17), and avoiding equipment sharing between cows within the farm (MI 01).

## Discussion

This study is a pioneer application of the best-worst scaling (BWS) method in analyzing farmers’ perspectives on the effectiveness of different MIs in the prevention and control of endometritis among zero-grazed dairy cows on smallholder farms. The approach has not been used previously in evaluating how dairy farmers perceive the effectiveness of different MIs for endometritis in dairy farms. The application of BWS method enabled the identification of 12 (60.0%) most effective MIs and 8 (40.0%) least effective MIs on smallholder farms practicing cut-and-carry feeding system in Rwanda.

As in the present study, the application of BWS with experts on opinion about the effectiveness of biosecurity measures on dairy farms informed livestock management practices in the United Kingdom [21]. In that study, preventing contact with neighbouring animals, and implementing rapid culling of persistently infected animals were rated the most effective biosecurity measures. In contrast, minimizing the number of visitors entering the farms, and avoiding equipment-sharing between farms were rated the least effective. Similarly, BWS was applied [26] to identify the effectiveness of intervention strategies for African swine fever in Western Europe. Findings suggest that the culling of all infected pigs and restricting movement for neighbouring farms were the most effective interventions to control the disease.

These findings reflect farmer experiences with MIs that effectively work for them. Farmer’s experiences with MIs indicate that farmers require a basket of choices of management practices from which to choose what suits their farming circumstances. Their practical skills can be integrated into extension strategies and veterinary service delivery to farmers and research attention to enhance herd health management [22].

In the current study, MIs (02, 04, and 10) related to equipment-sharing within the farm or with neighbouring farms and hygiene in cowshed were scored the most effective for preventing and controlling endometritis in dairy farms. The practice of sharing or borrowing farm equipment can be a media of disease and/or pathogen transmission [28]. This finding corroborates with the observations [29] in the United Kingdom that sharing equipment and materials between farms without appropriate biosecurity measures increases the risk of microorganisms contamination or disease transmission.

Farmer consideration that clean and dry cowshed (MI 10) and avoiding off-farm bedding materials (MI 04) are effective for prevention and control of endometritis has implications on hygiene practices. It is an indication that it is crucial to practice frequent removal of any soiled or damp bedding before adding fresh bedding materials. On the sampled farms, this was practiced on average twice a week. Unhygienic bedding materials and heavily soiled cattle shed are potential risk factors for transmission of causal microorganisms for disease in postpartum cows, of which endometritis is a prevalent fertility disease [30, 31]. Furthermore, high mastitis prevalence (76.2%) has been attributed [32] to inadequate biosecurity measures on zero-grazing dairy farms in Rwanda. Therefore, the implementation of biosecurity measures is essential to improving cow welfare as well as their production and fertility performance. This observation concurs with observations made in Ethiopia [33], in India [34], and in California, United States of America [35].

The MIs (05, 14, and 19) related to the control of breeding services scored highly for effectiveness. In the sampled farms, over half were using shared bulls for service without precautions for potential risks of disease transmission. Farmers used bull service because breeding bulls were readily available and affordable within the communities and about twice cheaper compared to artificial insemination (AI) ($3.3 vs. $5.6 per service). However, the bulls or the cows on heat are moved from one place to another for mating. The bulls and cows are not pre-screened for diseases before use. This way, the bull mating practice presents a risk of transmitting reproductive diseases, endometritis included as corroborated by finding [36] in Nyagatare district, Rwanda, and the United Kingdom dairy herds [21].

Farmers using AI services can be advised by their AI technicians about the advantages of selecting sires with easy-calving and can be assisted in choosing AI semen for their cows. The birth of a male calf may increase the risk of dystocia cases [37]. In such cases, giving calving assistance may also increase the likelihood of trauma and contamination of the reproductive tract and increase the risk of endometritis infections. Farmers can reduce the risk of dystocia by using sexed semen from sires with calving ease and low percent stillbirths to produce female calves [38].

The MIs (06 and 07) related to animal health intervention scored highly for effectiveness, implying that proper veterinary service delivery is essential for farmers in the prevention and control of endometritis disease [39]. In Brazil [40], metabolic disorders are important in the transition period because they predispose cows to reproductive disorders. Mastitis disease is also a significant risk factor for endometritis [41]. Proper veterinary service delivery facilitates prompt veterinary intervention for these risk factors.

However, in the present study, hardly half of the farmers accessed ANHS from VETs, CAHWs or LTHs. Basically, ANHS providers visit dairy herds at farmer’s request mostly when a health problem is noticed. In that case, the services offered target the diseases with commonly noticeable symptoms in dairy cows. Endometritis received less attention because the sampled farmers could not attribute the symptoms they observed to endometritis disease. This calls for capacity building program on endometritis diagnosis, extension advisory, and management on dairy farms to apply effective MIs for prevention and control of endometritis. This recommendation aligns with observation [20] in Uganda that improved extension service and advisory support in pre-and postpartum periods are effective ways to manage endometritis in the dairy herds.

Other MIs (03, 09, 17, and 20) that farmers considered highly effective for the prevention and control of endometritis related to reducing risks of contamination within and between farms. At high risk of contamination are fresh cows and cows with trauma in the reproductive tract because fresh cows are immunosuppressed, and housing them with mastitic cows exposes them to disease-causing pathogens [42]. Therefore, on-farm biosecurity measures are important to minimize the disease transmission in the dairy herds. Knowledge gaps might lead to widespread high-risk practices for both animals and humans [43]. From literature [42, 43], it is advisable to assist calving only when needed and always using gloved-hands, disinfecting calving equipment, and keeping cows in a clean cowshed and calving area to reduce trauma and contamination of female genital tract. Further, studies in France [44] and the United States of America [45] demonstrate the importance of good housing conditions, disinfection, and disease prevention in minimizing disease entry and spread within a dairy herd.

The adoption and implementation of the MIs that farmers perceived are most effective for the prevention and control of endometritis remain limited on the dairy farms in Rwanda. This situation applies as well to the United Kingdom [21] and Switzerland [46]. The reasons are likely related to some MIs being impractical to implement. This has to be addressed in in-depth research to enhance the practical application of MIs for the prevention and control of endometritis.

For the MIs that farmers considered the least effective for the prevention and control of endometritis, local contextual issues are likely to be at play. The sampled farmers had a low level of information about endometritis diagnosis and management; hence, could not have applied some of the MIs on their farms. Recent studies conducted in Switzerland [46] and Canada [47] showed that farmers’ awareness of the disease and a better understanding of the transmission of disease influence their perceptions on the effectiveness of biosecurity measures. Another study [48] with Indian farmers indicated that knowledge gaps about cattle diseases and how to prevent them limited the adoption of animal genetic improvement and health care practices.

In the study area, the VETs cover a wide area in the mountainous region with many farmers to attend to, which hinders prompt access in remote areas. CAHWs and LTHs could serve the remotely accessible areas for better and timely service delivery to farmers. In Indonesia [49] and Ghana [50], studies concluded that insufficient VETs and lack of capital hindered farmers from accessing prompt veterinary services and adopting and implementing biosecurity measures. In Canada, a study indicated that farmers who discussed biosecurity measure with a VET were more likely to perceive biosecurity measure as more effective than farmers who did not [47]. This is an indication that it is the best practice that farmers regularly consult with the ANHS providers on a plan of dairy herd health management.

Of the sampled farmers, about a third had a cowshed without a roof, and therefore, cows are not protected from environmental stresses such as the muddy floor in the zero-grazing unit. This condition favors bacterial contamination, where disease management practices are not implemented. It is because of the association between the lack of implementation of disease management practices and the high prevalence of mastitis in the dairy herds [32, 51, 52].

In Rwandan comprehensive wealth-ranking system criteria [53], the sample farmers were in the group classified as poor to very poor. Because they are the most vulnerable, resource-poor farmers, they have low capacity to implement the MIs [17]. This is corroborated [54] in Canadian dairy farms where a significant barrier to implementing prevention strategies for Johne’s disease was the cost to build facilities, hire more labor, and purchase the recommended equipment. In the present study farms, the land size owned was a resource constraint to farmers, necessitating practicing cut-and-carry feeding system in crowded cowshed units, with poor standards of hygiene. This condition exposes cows to bacterial contamination [55].

## Conclusion

This study has demonstrated the application of the BWS object case method in understanding the MIs that farmers consider are most effective in the prevention and control of endometritis disease in the dairy herds. The identified MIs can be prioritized for extension dissemination to farmers for effective prevention and control of endometritis disease. These are: avoiding sharing equipment with neighbouring farms, consulting animal health service provider about the treatment of endometritis cases, keeping cows in a clean and dry shed, and selecting sires based on calving ease. However, the MIs have to be applied in combination: no one of the MIs would be singly effective as there are multiple risk factors. In-depth research on these MIs is, however, necessary to enhance their practical application on smallholder dairy farms.

## Methods

### Study area

Data was collected from smallholder dairy farms with zero-grazed cows in Gasabo district of Rwanda (Additional file 1). The district is organized in 15 administrative sector units with 501 villages [56]. The study location lies at an altitude of 1800-m above sea level, with an annual mean temperature of 22 °C and a bimodal rainfall pattern averaging 1000 mm annually [1]. The district was chosen because of more prevalent dairy zero-grazing farms than the other districts [57]. Under smallholder dairy zero-grazing conditions, muddy conditions are prevailing, hygienic standards are low, herd health management plan is absent [32], risk exposure to bacterial disease infections is high, and the likelihood of endometritis disease infections is high.

A minimum sample of 150 farmers was computed as necessary for the study from the application of formula [58]:

$$ n=\frac{{Z_{1-\alpha /2}}^2p\left(1-p\right)}{d^2} $$

Where

*n* = sample size;

*Z*_1 − *α*/2_ = 95% level of confidence (1.96);

*p* = proportion of farmers aware of disease prevention and control, set at 11.0% [55];

*d*= desired absolute precision level, set at alpha 0.05.

### Survey design

This cross-sectional study recruited 154 farmers through a snowball sampling procedure. Sector animal resource officers identified the initial farmers in the sampling process. The researchers visited these farmers and, through snowball sampling, identified other farmers with the help of initial farmers. The initial farmers had to meet defined criteria. One, being aware of at least one a symptom of endometritis disease in cows (delayed conception, abortion or vaginal purulent or mucopurulent discharge) observed in the herd within the past 1 year. Two, granting written informed consent to participate in the study. Three, the farm is accessible physically. All the recruited farmers were visited between September 2018 and April 2019.

The study used the best-worst scaling (BWS) object case method to gather the perspectives of the farm owner about the effectiveness of MIs for endometritis prevention and control [21, 59]. This approach has been used for livestock disease management in many countries [21, 22, 26]. The BWS object case was applied to 20 MIs considered important in the prevention and control of endometritis (Table 3), based on the literature review [12, 14, 19, 60–63].

**Table 3:** Management interventions (MIs) examined in the study

MIs* codes	Management interventions (MIs)
1	Avoid equipment-sharing between cows within the farm
2	Avoid equipment-sharing with neighbouring farms
3	Avoid housing fresh cows with diseased cows or those with chronic illnesses such as mastitis
4	Avoid off-farm bedding materials and maintain adequate bedding materials per cow
5	Avoid sharing or hiring a breeding bull
6	Consult ANHS* provider about the treatment and prevention of mastitis and metabolic diseases
7	Consult ANHS* providers about the treatment of endometritis positive cases
8	Cull of persistently endometritis positive cows
9	Disinfect equipments used in calving assistance before and after use
10	Keep the cows in a clean and dry shed
11	Maintain adequate feeding per cow
12	Maintain a clean transition cow housing
13	Maintain regular contact with ANHS* providers for advisory support on endometritis prevention in dairy farm
14	Select sires based on calving ease
15	Select sires based on low percent stillbirths
16	Remove fetal membranes immediately after passing
17	Use gloves during calving assistance
18	Use an artificial insemination service
19	Use a sexed semen during artificial insemination service
20	Wash the hands and udder before each milking

*MIs Management interventions, *ANHS* Animal health service

In this study, MIs was defined as an action that reduced or targeted risk factors for endometritis in the dairy herds. The effectiveness of MIs was the extent to which the MIs prevented or controlled endometritis-causing agents on-farm.

The check-list for best-worst scaling choice was designed in Sawtooth Software (Version 8) [64] with defined criteria: (i) each MI appeared in an equal number of times in a different choice card; (ii) each MI paired with any other MI an equal number of times; (iii) each MI appeared four times in total across all choice cards and (iv) the order of MIs within each choice card was randomly assigned [64, 65].

The questionnaire was designed in such a way that each dairy farmer had to respond to a total of 16 choice cards of five MIs each. The questionnaire was developed in English (Additional file 2), and the researchers conducted the interviews in the local language (Kinyarwanda). The questionnaire was pre-tested on 30 farmers to ensure the objective of the study is clear, to determine the time needed to complete the survey per farmer and the obstacle that could arise, and to improve the clarity of the questions to respondents [66]. The 30 farmers used in the pre-testing survey were not part of the farmers recruited for the study being reported in this paper.

Subsequently, for each choice card, the farmer was asked to choose first the most effective and then the least effective MI for endometritis prevention and control (Table 4). To increase variation and combination of MIs across dairy farmers, the order of 16 choice cards was randomly assigned for each dairy farmer using Microsoft Office Excel (version 2016) [59]. The socio-demographic characteristics of each participating farm and farmer were recorded.

**Table 4:** Example of an effectiveness best-worst scaling choice card used in the study

MIs* codes	Management interventions (MIs)	Most effective	Least effective
2	Avoid equipment-sharing with neighbouring farms	☐	☐
19	Use a sexed semen during artificial insemination service	☐	☐
6	Consult ANHS* providers about the treatment and prevention of mastitis and metabolic diseases	☐	☐
15	Select sires based on low percent stillbirths by referring to dairy sires catalogue	☐	☐
10	Keep cows in a clean and dry shed	☐	☐

*MIs Management interventions, *ANHS* Animal health service provider

### Statistical analysis

Data analysis of the best-worst choices were performed using two approaches: (i) best-worst percentages for the number of times each MI was selected as most effective, least effective or not chosen across a sequence of 16 choice cards divided by the availability of each MI; and (ii) best–worst score as the standardized score for each MI [59, 67]. The score was computed as the difference between the number of times chosen as the most and least effective divided by the availability of each MI [59, 68]. The availability of each MI was computed as the number of times it has appeared in total across the 16 choice cards multiplied by the sample size [59, 69].

The computed standardized score indicates the effectiveness of MI on a scale from − 1.0 to + 1.0. A positive score (+ 1.0) indicates that the MI was chosen as most effective more often than as least effective. In contrast, the negative score (− 1.0) suggests that the MI was chosen as least effective more often than as most effective [27, 59]. Thus, the higher the score, the more the MI was effective. In this study, different MIs were categorized into four-group endometritis prevention and control plan relating to (i) equipment-sharing between cows within farm and/or with neighbouring farms and hygiene in cowshed, (ii) control of breeding services, (iii) animal health interventions, and (iv) reduce the risks of contamination within and between farms.

Frequency distribution describing farmer characteristics was generated from cross-tabulation and frequency tested with Chi-square test statistics. Hypothesis testing was with the Non-parametric Kruskal-Wallis H test for whether the source of ANHS providers, education level, or poverty level differ by farmer’s choices. A Binomial test was used to analyze the significance of the differences in cattle shed flooring (earthen or concrete), cattle shed types (covered with or without a roof), breeding services (artificial insemination or bull) and herds recording (not practiced or incomplete). All statistical analyses were performed in IBM SPSS Statistics 22.0 for Windows [70], and the statistical significance level was set at alpha < 0.05.

## Supplementary information

**Additional file 1.** Map of the study area. The additional file 1 illustrates the map for study setting, and authors generated it. During this study, GPS data were collected on the location of each farmer’s household using GPS eTrex 10 Garmin. QGIS version 2.18.20-Las Palmas software was used to produce the map depicted in additional file 1 based on the GPS data. Attribution: GIS Layers: Humanitarian Data Exchange. https://www.data.humdata.org.

**Additional file 2.** Questionnaire used for the farmers’ interviews. The additional file 2 illustrates the questionnaire for the research on the perception of farmers about Endometritis prevention and control measures for zero-grazed dairy cows on smallholder farms in Rwanda.

## Data Availability

The questionnaire used is available (Additional file 2). Map of the study area is available (Additional file 1). The datasets used and/or analyzed during the current study are available from the corresponding author on reasonable request.

## Abbreviations

ANHS: Animal Health Service
BWS: Best-Worst Scaling
CAHWs: Community-Based Animal Health Workers
GDP: Gross Domestic Product
LTHs: Local Traditional Herbalists
MIs: Management Interventions
SPSS: Statistical Package for the Social Sciences
VETs: Veterinarians
