# Supplementary figures and images for: Perception of farmers about endometritis prevention and control measures for zero-grazed dairy cows on smallholder farms in Rwanda
Source: BMC Vet Res. 2020 Jun 5;16:175. doi: 10.1186/s12917-020-02368-6 (PMC7275537; doi:10.1186/s12917-020-02368-6)

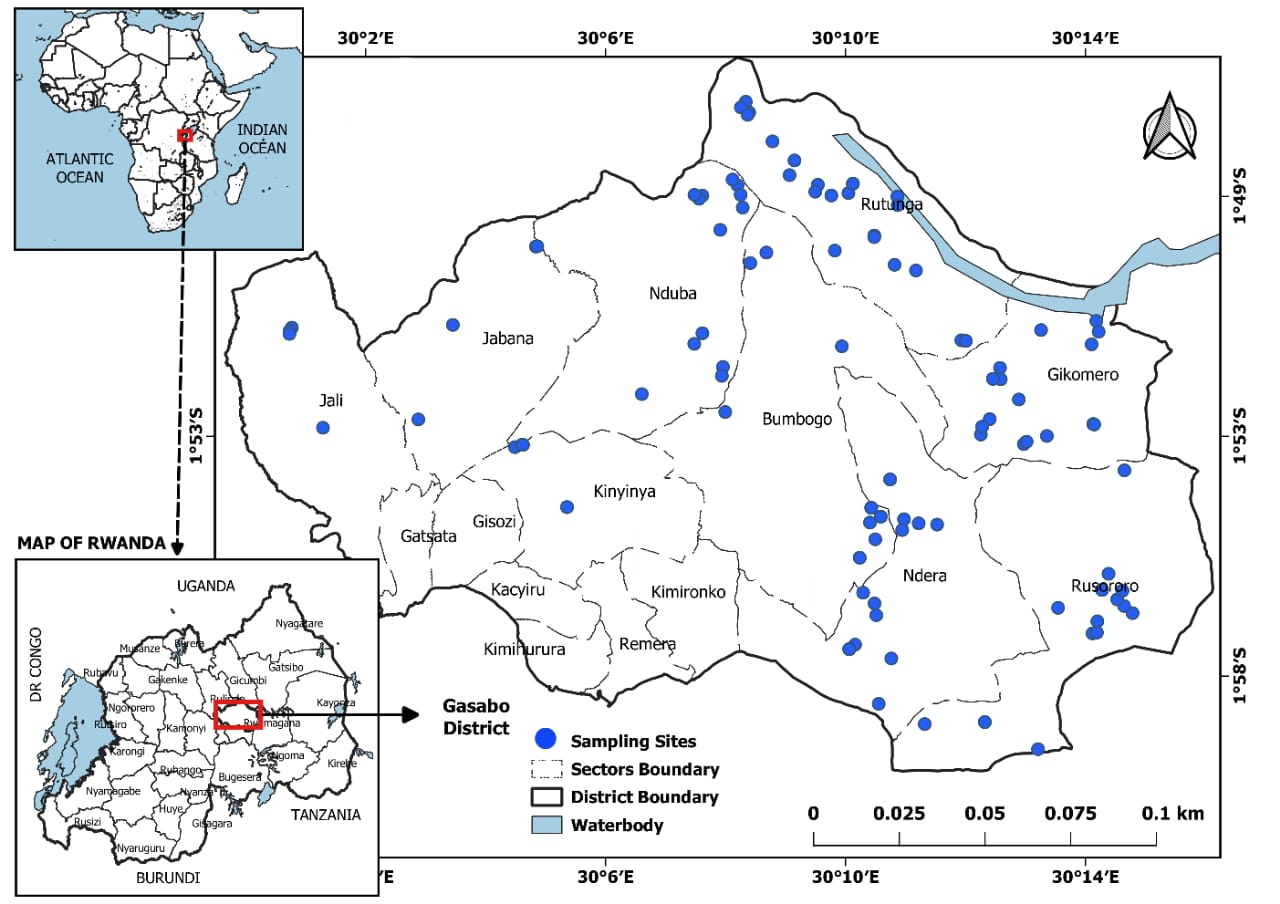

Supplement: Supplementary file 1 — Additional file 1. Map of the study area. The additional file 1 illustrates the map for study setting, and authors generated it. During this study, GPS data were collected on the location of each farmer’s household using GPS eTrex 10 Garmin. QGIS version 2.18.20-Las Palmas software was used to produce the map depicted in additional file 1 based on the GPS data. Attribution: GIS Layers: Humanitarian Data Exchange. https://www.data.humdata.org. [file 12917_2020_2368_MOESM1_ESM.jpg]
