# Supplementary material for: Perception of farmers about endometritis prevention and control measures for zero-grazed dairy cows on smallholder farms in Rwanda
Source: BMC Vet Res. 2020 Jun 5;16:175. doi: 10.1186/s12917-020-02368-6 (PMC7275537; doi:10.1186/s12917-020-02368-6)
Supplement: Supplementary file 2 — Additional file 2. Questionnaire used for the farmers’ interviews. The additional file 2 illustrates the questionnaire for the research on the perception of farmers about Endometritis prevention and control measures for zero-grazed dairy cows on smallholder farms in Rwanda. [file 12917_2020_2368_MOESM2_ESM.pdf]

Questionnaire for the research on perception of farmers about Endometritis prevention and control measures for zero-grazed dairy cows on smallholder farms in Rwanda

---

Dear Sir/Madam,

The above mentioned study is led by Mr. Pascal Nyabinwa from Egerton University-Njoro Campus, Kenya. Your information and experiences will be used solely for the academic purpose(s) and will remain confidential. The interview will take approximately one hour and your participation is cornerstone to the success of this study. We are kindly asking for your consent to be part of the study.

Farmer consent obtained / __ / 1.Yes 2. No

Name of participant ________________________________ Signature________________

Date /___/ __ __/__ __ __ __/ Thank you.

Farm No. /____ ____ ____/

Questionnaire No. / ___ ___ ___ /

Date (*dd/mm/yy*) /____/____/____/

Enumerator name /________________________________ / Enumerator Code / __ __ /

Enumerator contact /__________________________________ /

Reviewed/supervised by /_______________________________ /

SECTION A: General information on farmer and farm management

A.1. Information on farmer

1. Respondent's name / ___________________________________ /
2. Tel. number / _________________________ /
3. Gender of the respondent / ____ / *1 = Male 2 = Female*
4. Age of the respondent / ____ ____ / years
5. Household size (persons) / ____ ____ /
6. Category in national wealth ranking system (Ubudehe) / ______ /
7. Education level /__/ *1 = No schooling 2 = Primary school 3 = Secondary school 4= University 5 = Other (specify) / __________________ /*
8. Dairy farming experience in years / ___ ___ /
9. Farmer's location *(i) Village / ________________ / (ii) Cell / _______________ /*
   (iii) Sector / _________________ / (iv) GPS coordinates: S /______________/
   E /_____________/

A.2. Information on farm management

10. Population (number) of cattle kept on the farm by genotypes

1 = Indigenous cattle / _ _ /, 2 = Dairy crossbreds / __ __ /, 3 Dairy pure breeds / __ _/

11. What total land size do you farm now in acres? / ____ ____ ___ /
12. Herd records keeping / ______ / *1 = Complete, 2 = Incomplete, 3 = Not practiced*
13. Breeding service used on the farm / _________ / *1 = Artificial Insemination Service, 2 = Bull Service, 3 = Both*
14. Breeding service cost ($), *Artificial insemination service* / __ /, *Bull service* / __ / ($1 = Rwf 920)
15. Source of animal health services / ___ / *1 = Veterinary, 2 = Community-based animal health workers, 3 = Local traditional herbalists*
16. Do you cut-and-carry fodder and crop residues to your animals? / _____ / *1 = Yes, 2 = No*
17. Housing floor types / ___ / *1 = Concrete, 2 = Wooden, 3 = Earthen*
18. Type of cattle shed / ___ / *1 = with a roof, 2 = Without a roof*
19. Do you use bedding materials in cowshed? / ___ / *1 = Yes, 2 = No*

If Yes, indicate which bedding materials are used / ___ ___ / 1 = *Sand, 2 = natural green grasses, 3 = corn*, *4 = wood shaving, 5 = leftover or waste feeds from feeding troughs, 6 = hay, 7 = Sawdust, 8 = straw*

20. Frequency of removing any soiled or damp bedding before adding fresh bedding materials / ___ / *1= Daily basis, 2 = Once per week, 3 = Twice per week, 4 = Once per month , 5 = Twice per month, 6 = Other (specify)*

SECTION B: Effectiveness of management interventions for endometritis in smallholder zero-grazed dairy farms in Rwanda

B.1. Farmer perception of endometritis signs in cows in a herd

Endometritis is a postpartum disease characterized by an inflammation of the uterine endometrium 21-90 days postpartum period. According to its signs: (i) white or whitish-yellow mucopurulent vaginal discharge comes out when the animal lies down, urinates or defecates and visible externally on the tail, perineum and vulva; (ii) mucopurulent discharge at the time of oestrus, (iii) repeat breeding and (iv) failure to conception

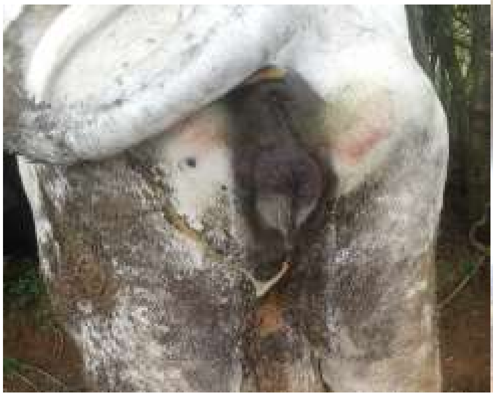
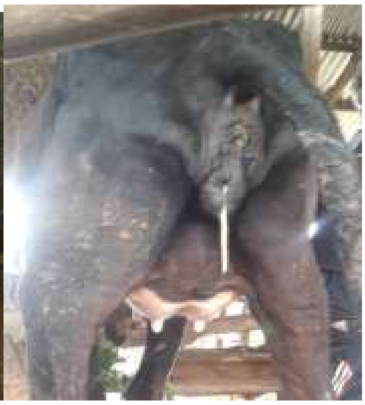
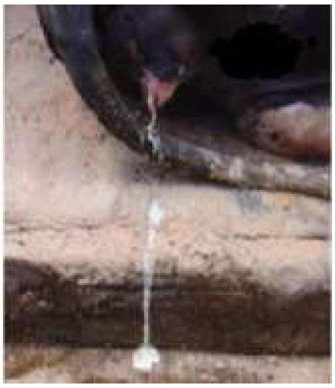

Did you observe such signs in cows in a herd for the past one year? / ___ /<br>1 = yes, 2 = no

B.2. Farmer perceived effective management intervention for endometritis among zero-grazed dairy cows on smallholder farms

For each best-worst scaling choice card (BWSCC1), please pick the most effective and the least effective management interventions for endometritis prevention and control on dairy farms.

BWSCC-1

MIs codes	Management interventions	Most effective	Least effective
7	Consult ANHS provider about the treatment of endometritis positive case	☐	☐
12	Maintain clean transition cow housing	☐	☐
1	Avoid equipment-sharing between cows into the farm	☐	☐
5	Avoid sharing or hiring breeding bull	☐	☐
20	Wash the hands and udder before each milking	☐	☐

BWSCC-2

MIs codes	Management interventions	Most effective	Least effective
16	Remove fetal membranes immediately after passing	☐	☐
9	Disinfect equipment used in calving assistance before and after use	☐	☐
8	Cull of persistently endometritis positive cows	☐	☐
13	Maintain regular contact with ANHS provider for advisory support on endometritis prevention in dairy farm	☐	☐
3	Avoid housing fresh cows with diseased cows or those with chronic illnesses such as mastitis	☐	☐

BWSCC-3

MIs codes	Management interventions	Most effective	Least effective
2	Avoid equipment-sharing with neighbouring farms	☐	☐
19	Use a sexed semen during artificial insemination service	☐	☐
6	Consult ANHS provider about the treatment and prevention of mastitis and metabolic diseases	☐	☐
15	Select sires based on low percent stillbirths by referring to sires catalogue	☐	☐
10	Keep cows in a clean and dry shed	☐	☐

BWSCC-4

MIs codes	Management interventions	Most effective	Least effective
18	Use an artificial insemination service	☐	☐
4	Avoid off-farm bedding materials and maintain adequate bedding materials per cow	☐	☐
14	Select sires based on calving ease by referring to dairy sires catalogue	☐	☐
11	Maintain adequate feeding per cow	☐	☐
17	Use gloves during calving assistance	☐	☐

BWSCC-5

MIs codes	Management interventions	Most effective	Least effective
1	Avoid equipment-sharing between cows into the farm	☐	☐
13	Maintain regular contact with ANHS provider for advisory support on endometritis prevention in dairy farm	☐	☐
19	Use a sexed semen during artificial insemination service	☐	☐
11	Maintain adequate feeding per cow	☐	☐
16	Remove fetal membranes immediately after passing	☐	☐

BWSCC-6

MIs codes	Management interventions	Most effective	Least effective
10	Keep cows in a clean and dry shed	☐	☐
8	Cull of persistently endometritis positive cows	☐	☐
20	Wash the hands and udder before each milking	☐	☐
17	Use gloves during calving assistance	☐	☐
3	Avoid housing fresh cows with diseased cows or those with chronic illnesses such as mastitis	☐	☐

BWSCC-7

MIs codes	Management interventions	Most effective	Least effective
18	Use an artificial insemination service	☐	☐
2	Avoid equipment-sharing with neighbouring farms	☐	☐
5	Avoid sharing or hiring breeding bull	☐	☐
9	Disinfect equipment used in calving assistance before and after use	☐	☐
15	Select sires based on low percent stillbirths by referring to sires catalogue	☐	☐

BWSCC-8

MIs codes	Management interventions	Most effective	Least effective
4	Avoid off-farm bedding materials and maintain adequate bedding materials per cow	☐	☐
12	Maintain clean transition cow housing	☐	☐
6	Consult ANHS provider about the treatment and prevention of mastitis and metabolic diseases	☐	☐
7	Consult ANHS provider about the treatment of endometritis positive case	☐	☐
14	Select sires based on calving ease by referring to dairy sires catalogue	☐	☐

BWSCC-9

MIs codes	Management interventions	Most effective	Least effective
13	Maintain regular contact with ANHS provider for advisory support on endometritis prevention in dairy farm	☐	☐
10	Keep cows in a clean and dry shed	☐	☐
12	Maintain clean transition cow housing	☐	☐
18	Use an artificial insemination service	☐	☐
6	Consult ANHS provider about the treatment and prevention of mastitis and metabolic diseases	☐	☐

BWSCC-10

MIs codes	Management interventions	Most effective	Least effective
8	Cull of persistently endometritis positive cows	☐	☐
11	Maintain adequate feeding per cow	☐	☐
7	Consult ANHS provider about the treatment of endometritis positive case	☐	☐
15	Select sires based on low percent stillbirths by referring to sires catalogue	☐	☐
17	Use gloves during calving assistance	☐	☐

BWSCC-11

MIs codes	Management interventions	Most effective	Least effective
4	Avoid off-farm bedding materials and maintain adequate bedding materials per cow	☐	☐
20	Wash the hands and udder before each milking	☐	☐
16	Remove fetal membranes immediately after passing	☐	☐
2	Avoid equipment-sharing with neighbouring farms	☐	☐
1	Avoid equipment-sharing between cows into the farm	☐	☐

BWSCC-12

MIs codes	Management interventions	Most effective	Least effective
19	Use a sexed semen during artificial insemination service	☐	☐
9	Disinfect equipment used in calving assistance before and after use	☐	☐
14	Select sires based on calving ease by referring to dairy sires catalogue	☐	☐
3	Avoid housing fresh cows with diseased cows or those with chronic illnesses such as mastitis	☐	☐
5	Avoid sharing or hiring breeding bull	☐	☐

BWSCC-13

MIs codes	Management interventions	Most effective	Least effective
12	Maintain clean transition cow housing	☐	☐
11	Maintain adequate feeding per cow	☐	☐
2	Avoid equipment-sharing with neighbouring farms	☐	☐
3	Avoid housing fresh cows with diseased cows or those with chronic illnesses such as mastitis	☐	☐
15	Select sires based on low percent stillbirths by referring to sires catalogue	☐	☐

BWSCC-14

MIs codes	Management interventions	Most effective	Least effective
6	Consult ANHS provider about the treatment and prevention of mastitis and metabolic diseases	☐	☐
9	Disinfect equipment used in calving assistance before and after use	☐	☐
17	Use gloves during calving assistance	☐	☐
1	Avoid equipment-sharing between cows into the farm	☐	☐
14	Select sires based on calving ease by referring to dairy sires catalogue	☐	☐

BWSCC-15

MIs codes	Management interventions	Most effective	Least effective
13	Maintain regular contact with ANHS provider for advisory support on endometritis prevention in dairy farm	☐	☐
5	Avoid sharing or hiring breeding bull	☐	☐
4	Avoid off-farm bedding materials and maintain adequate bedding materials per cow	☐	☐
8	Cull of persistently endometritis positive cows	☐	☐
19	Use a sexed semen during artificial insemination service	☐	☐

BWSCC-16

MIs codes	Management interventions	Most effective	Least effective
10	Keep cows in a clean and dry shed	☐	☐
16	Remove fetal membranes immediately after passing	☐	☐
7	Consult ANHS provider about the treatment of endometritis positive case	☐	☐
18	Use an artificial insemination service	☐	☐
20	Wash the hands and udder before each milking	☐	☐

Thank you for your participation.
